# Prevalence and factors associated with metabolic syndrome in an urban population of adults living with HIV in Nairobi, Kenya

**DOI:** 10.11604/pamj.2018.29.90.13328

**Published:** 2018-01-30

**Authors:** Catherine Nduku Kiama, Joyce Njeri Wamicwe, Elvis Omondi Oyugi, Mark Odhiambo Obonyo, Jane Githuku Mungai, Zeinab Gura Roka, Ann Mwangi

**Affiliations:** 1 Moi University School of Public Health, Eldoret, Kenya; 2 Field Epidemiology and Laboratory Training Program, Ministry of Health, Nairobi, Kenya; 3 National AIDS and STI Control Program, Ministry of Health, Nairobi, Kenya

**Keywords:** Metabolic syndrome, prevalence, associated factors, HIV

## Abstract

**Introduction:**

Metabolic syndrome affects 20-25% of the adult population globally. It predisposes to cardiovascular disease and Type 2 diabetes. Studies in other countries suggest a high prevalence of metabolic syndrome among HIV-infected patients but no studies have been reported in Kenya. The objective of this study was to assess the prevalence and factors associated with metabolic syndrome in adult HIV-infected patients in an urban population in Nairobi, Kenya.

**Methods:**

In a cross-sectional study design, conducted at Riruta Health Centre in 2016, 360 adults infected with HIV were recruited. A structured questionnaire was used to collect data on socio-demography. Blood was collected by finger prick for fasting glucose and venous sampling for lipid profile.

**Results:**

Using the harmonized Joint Scientific Statement criteria, metabolic syndrome was present in 19.2%. The prevalence was higher among females than males (20.7% vs. 16.0%). Obesity (AOR = 5.37, P < 0.001), lack of formal education (AOR = 5.20, P = 0.002) and family history of hypertension (AOR = 2.06, P = 0.029) were associated with increased odds of metabolic syndrome while physical activity (AOR = 0.28, P = 0.001) was associated with decreased odds.

**Conclusion:**

Metabolic syndrome is prevalent in this study population. Obesity, lack of formal education, family history of hypertension, and physical inactivity are associated with metabolic syndrome. Screening for risk factors, promotion of healthy lifestyle, and nutrition counselling should be offered routinely in HIV care and treatment clinics.

## Introduction

Metabolic syndrome is a cluster of risk factors in an individual that increase the risk of developing cardiovascular disease and Type 2 diabetes [1-3]. Patients with metabolic syndrome are more likely to suffer heart attack, stroke and cardiovascular mortality [4-7]. The global burden of cardiovascular disease and diabetes is increasing, with the biggest burden in lower and middle income countries. This is expected to rise substantially over the next two decades [8]. Human immunodeficiency virus (HIV) and use of antiretroviral treatment (ART) has led to an increase in cases of metabolic syndrome. HIV natural progression of disease is known to cause lipid abnormalities [9]. ART has resulted in improved quality of life, survival to older ages for those living with HIV and emergence of metabolic adverse events related to use of ART; dyslipidemia, changes in body composition, insulin resistance and glucose intolerance [10-12]. Prospective analysis of patients initiated on ART demonstrated 8.4% increase in prevalence of metabolic syndrome at 48 weeks of antiretroviral treatment [13]. It is estimated that 20-25% of adults globally have metabolic syndrome [14]. Prevalence in sub-Saharan Africa ranges from 0 -50% or higher and differs from population to population [15, 16]. Studies conducted in the general population from urban centres in Kenya reported the prevalence as 25.6% (several urban centres) and 34.6% (one urban centre) [17, 18]. Among people living with HIV (PLHIV), studies conducted in several African countries report prevalence of up to 60.6% [19-22]. According to the 2014 Kenya HIV Estimates Report, Kenya has 1.6 million PLHIV and 101,900 new HIV infections annually. There is limited data on metabolic syndrome in this population in Kenya since screening at the ART clinics is not conducted routinely. The burden of metabolic syndrome among PLHIV in Kenya is not well understood. We sought to find out the differences in socio-demographics and risk factors between the HIV-infected patients with metabolic syndrome and those without the syndrome. The aim of the study was to assess the prevalence and factors associated with metabolic syndrome in HIV-infected patients in a predominantly urban population in Nairobi, Kenya.

## Methods

**Study design and study population** : This was a hospital based cross-sectional study. The study population was sampled HIV positive patients attending the Riruta Health centre ART clinic. The eligibility criteria for the study participants were; patients aged ≥18 years, visiting the ART clinic with a scheduled appointment for routine follow up, enrolled in care (either on prophylaxis or ART) at least 6 months prior to the study and fasted overnight (for 12 hours). We excluded clients with missing medical records, those on interrupted treatment, those visiting due to an acute illness and pregnant women. The Health centre serves as an outpatient facility offering HIV care and treatment amongst other services. It is situated in Dagoretti within the capital city of Nairobi and serves a predominantly urban population. It is considered a high volume HIV care and treatment site with an average of 100 HIV-infected patients scheduled for a clinic visit each day and a total of 3,027 patients active and on follow up at the start of the study. The study was conducted between May and June 2016.

**Sample size calculation and selection of study participants**: A sample size of 348 was estimated using Cochran's formula (1977) with the following assumptions; 34.6% prevalence of metabolic syndrome among the general population in Nairobi [7], 5% precision and a 95% confidence interval. The study participants were selected using random systematic sampling of HIV-infected patients attending routine clinic visits. The Health centre receives 100 HIV patients, on average, per day. We first allocated each patient a number from 1 to 100 and since we needed to enroll at least 20 patients per day, we selected every fifth patient to be included in the study until the required sample size was reached. With the help of the ART clinic staff, the selected patient was contacted on phone and advised to have supper as the last meal and skip breakfast on the clinic visit day in order to collect some blood for tests.

**Operational definitions**: metabolic syndrome was defined according to the harmonized Joint Scientific Statement criteria [23], which is the presence of any three of the five following risk factors; (1) waist circumference > 94cm for men or > 80cm for women, (2) triglycerides > 1.7 mmol/L or specific treatment for this abnormality, (3) HDL-Cholesterol < 1.03 mmol/L for men or < 1.29 mmol/L for women or specific treatment for this abnormality, (4) elevated blood pressure > 130/85 mmHg or treatment of previously diagnosed hypertension and (5) elevated fasting glucose > 5.6 mmol/L or treatment of previously diagnosed diabetes. Waist circumference was used to detect presence of abdominal obesity by measuring abdominal fat.

**Data collection**: The questionnaire was prepared in English. The clinician attending to the patient confirmed that the patient had supper as their last meal, obtained informed consent and administered the questionnaire. We collected socio-demographic and risk factor information through face to face interviews. The socio-demographic variables collected were; age, gender, highest level of education, marital status, religion, occupation and residence. Risk factor information collected were; family history (of hypertension, diabetes, asthma, cancers, cardiovascular disease), previous diagnosis (of diabetes, hypertension, lipid abnormalities), physical inactivity, fast food diet, stress, obesity, smoking and alcohol use. The clinician also carried out the physical examination and abstracted clinical information from the patients' files. Clinical information collected included; WHO staging of HIV disease, history of ART use, number of years on ART and last CD4 count. At the end of the visit, blood sugar testing was performed by the clinician at the point of care according to the Accu-Check glucometer manufacturer's specifications. About 4.5 mls of venous blood was collected from the patients at the facility laboratory by a trained phlebotomist, labelled with a unique number, age and sex and then shipped to the National Public Health Laboratory for lipid profile testing. Lipid profile testing was done using the Cobas C111 analyzer. At the end of each day, data was entered into Epi info v.7.14 (CDC TM) and stored in Microsoft Access database on a personal computer.

**Data analysis**: The data was analyzed using Epi info v.7.14 (CDC TM) software. Univariate analysis was performed and descriptive statistics reported using frequency distribution, proportions and means for socio-demographic and clinical variables. The prevalence of metabolic syndrome (95% Confidence Interval) was determined based on the definition criteria used in the study. Descriptive statistics were summarized for females and males to test if there was a statistical difference between the two groups. Bivariate analysis of risk factors undertaken using Odds Ratio as a measure of association and the statistical significance checked using Confidence Interval and Chi square test with P < 0.05 considered statistically significant. Binary logistic regression using the forward additive method was performed to identify the risk factors and to measure the variation in association between the independent and dependent variables. Factors with P < 0.25 from bivariate analysis were included in logistic regression to arrive at the final model of independent predictors of metabolic syndrome.

**Ethical considerations**: Written informed consent was obtained from all the study participants. The study protocol was reviewed and approved by Moi Teaching and Referral Hospital Institutional Ethics and Research Committee (Approval No. 0001531).

## Results

**Figure 1:**
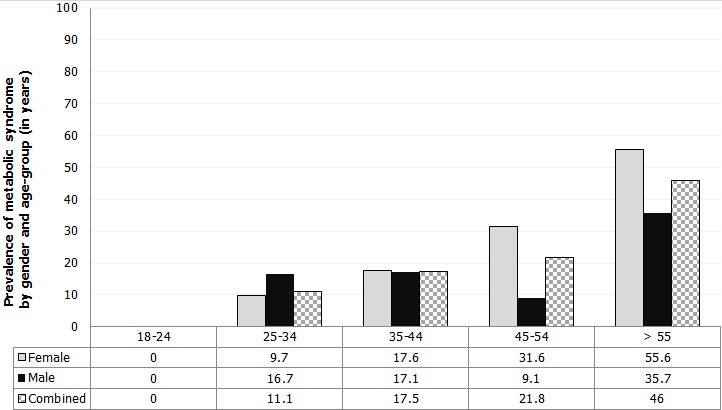
Prevalence of metabolic syndrome among adult HIV-infected patients by gender and age-group, Riruta Health Centre, 2016

A total of 360 patients were recruited with 66.9% being female (Table 1). Using the harmonized Joint Scientific Statement criteria, the overall prevalence of metabolic syndrome was 19.2% (95% C.I 15.3-23.7), higher in females than males. There was no case of metabolic syndrome observed among patients aged 18-24 years. The prevalence increased with age with patients older than 55 years having the highest prevalence (Figure 1). The prevalence of metabolic syndrome components in our study participants was; abdominal obesity 51.1%, low HDL-Cholesterol 32.5%, systolic hypertension 32.5%, hyperglycaemia 21.4% and hypertriglyceridemia 14.2%. The prevalence of abdominal obesity and low HDL-Cholesterol was higher among the female patients. However, the prevalence of hypertension, hyperglycaemia and hypertriglyceridemia was higher among the male patients (Table 2). There were either few or no patients aged 18-24 years with any of the metabolic syndrome components. On bivariate analysis, obesity (BMI > 30kg/m^2^), informal education, age ≥ 45 years and family history of hypertension were associated with increased odds of metabolic syndrome. Patients on antiretroviral treatment and those who were physically active had decreased odds of metabolic syndrome. Metabolic syndrome was not associated with gender, employment status, history of cardiovascular disease and diabetes in the family, smoking, alcohol use, consumption of fast foods and stress (Table 3). On multivariate analysis, there was a positive association between obesity, lack of formal education, family history of hypertension and occurrence of metabolic syndrome. Patients who were physically active had reduced odds of having metabolic syndrome (Table 4).

**Table 1:** Socio-demographic and clinical characteristics of adult HIV-infected patients, Riruta Health Centre, 2016

Characteristic	CombinedNo. (%)	FemaleNo. (%)	MaleNo. (%)	*p-*value
*N*	360	241	119	
Age-group (years)				
18-24	11 (3.1)	9 (81.8)	2 (18.2)	0.285
25-34	90 (25.0)	72 (80.0)	18 (20.0)	0.002
35-44	126 (35.0)	85 (67.5)	41 (32.5)	0.881
45-54	101 (28.1)	57 (56.4)	44 (43.6)	0.008
>55	32 (9.9)	18 (56.2)	14 (43.8)	0.177
Highest level of education				
No education	18 (5.0)	15 (83.3)	3 (16.7)	0.129
Primary incomplete	82 (22.8)	61 (74.4)	21 (25.6)	0.103
Primary complete	109 (30.3)	75 (68.8)	34 (31.2)	0.617
Secondary/ Tertiary	151 (41.9)	90 (59.6)	61 (40.4)	0.012
Marital status				
Never Married	37 (10.3)	31 (83.8)	6 (16.2)	0.021
Married	190 (52.8)	93 (48.9)	97 (51.1)	<0.001
Living together/Cohabiting	5 (1.4)	5 (100.0)	0 (0.0)	0.114
Divorced/ Separated	80 (22.2)	70 (87.5)	10 (12.5)	<0.001
Widowed	48 (13.3)	42 (87.5)	6 (12.5)	0.001
Religion				
Muslim	9 (2.5)	7 (80.0)	0 (0.0)	0.060
Protestant/ Other Christian	266 (73.9)	186 (69.9)	80 (30.1)	0.043
Roman Catholic	75 (20.8)	42 (56.0)	33 (44.0)	0.024
No religion	4 (1.1)	0 (0.0)	4 (100.0)	0.004
Other	6 (1.7)	6 (100.0)	0 (0.0)	0.082
Occupation				
Employed/Self Employed	198 (55.0)	125 (63.1)	73 (36.9)	0.089
Student	3 (0.8)	2 (66.7)	1 (33.3)	0.992
Unemployed	54 (15.0)	48 (88.9)	6 (11.1)	<0.001
Unskilled labor	105 (29.2)	66 (62.3)	39 (37.7)	0.289
Residence				
Rural	3 (0.8)	1 (33.3)	2 (66.7)	0.215
Urban	357 (99.2)	240 (67.2)	117 (32.8)	0.215
On Antiretroviral treatment (ART)				
Yes	355 (98.6)	236 (66.5)	119 (33.5)	0.114
No	5 (1.4)	5 (100.0)	0 (0.0)	0.114
WHO stage				
1	334 (92.8)	221 (66.2)	113 (33.8)	0.263
2	7 (1.9)	6 (85.7)	1 (14.3)	0.285
3	6 (1.7)	4 (66.7)	2 (33.3)	0.992
4	1 (0.3)	1 (100.0)	0 (0.0)	0.484
Number of years on ART mean ± SD	4.5 ± 3.2	4.5 ± 3.1	4.6 ± 3.4	0.781
CD4 Count mean ± SD	357.8 ± 223.6	393.2 ± 234.9	286.5 ±179.6	<0.0001

A total of 12 patients (3.3%): 9 female and 3 male did not have a WHO stage recorded in their files

**Table 2:** Prevalence of metabolic syndrome components among adult HIV-infected patients, Riruta Health Centre, 2016

Metabolic syndrome components	CombinedNo. (%)	FemaleNo. (%)	MaleNo. (%)	*p-*value
*N*	360	241	119	
*Metabolic syndrome*	69 (19.2)	50 (20.7)	19 (16.0)	0.280
High waist circumference	184 (51.1)	141 (58.5)	43 (36.1)	< 0.001
Low HDL – Cholesterol	117 (32.5)	88 (36.5)	29 (24.4)	0.021
Systolic Blood pressure ≥ 130mmHg	117 (32.5)	71 (29.5)	46 (38.7)	0.080
Glycaemia ≥ 5.6 mmol/L	77 (21.4)	50 (20.7)	27 (22.7)	0.675
Triglycerides ≥ 1.7 mmol/L	51 (14.2)	31 (12.9)	20 (16.8)	0.313

Waist circumference cut off ≥ 94 cm for men or ≥ 80 cm for women

Low HDL – Cholesterol was defined as < 1.03 for men or < 1.29mmol/L for women

**Table 3:** Bivariate analysis of risk factors for metabolic syndrome in adult HIV-infected patients, Riruta Health Centre, 2016

Factors	With metabolicSyndromen = 69	Without metabolicsyndromen = 291	Crude OR(95% C.I)	*p*-value
Female				
Yes	50	191	1.38 (0.77 – 2.46)	0.278
No	19	100		
≥ 45 years				
Yes	37	96	2.35 (1.38 – 4.00)	0.001
No	32	195		
Informal employment				
Yes	64	5	2.53 (0.97 – 6.61)	0.058
No	243	48		
Informal education				
Yes	9	9	4.70 (1.79 – 12.34)	< 0.001
No	60	282		
On Antiretroviral treatment				
Yes	66	289	0.15 (0.02 – 0.93)	0.051
No	3	2		
Family history of cardiovascular disease				
Yes	5	10	2.20 (0.73 – 6.64)	0.154
No	64	281		
Family history of diabetes mellitus				
Yes	14	49	1.26 (0.65 – 2.44)	0.498
No	55	242		
Family history of hypertension				
Yes	25	63	2.06 (1.17 – 3.62)	0.011
No	44	228		
History of smoking				
Yes	6	36	0.675 (0.27 – 1.67)	0.393
No	63	255		
Alcohol use				
Yes	6	22	1.16 (0.45 – 2.99)	0.752
No	63	269		
Physical activity				
Yes	10	115	0.26 (0.13 – 0.53)	< 0.001
No	59	176		
Eating fast foods				
Yes	36	145	1.10 (0.65 – 1.86)	0.726
No	33	146		
Stress				
Yes	30	126	1.00 (0.59 – 1.71)	0.978
No	39	165		
Obesity				
Yes	27	32	5.20 (2.84 – 9.55)	< 0.001
No	42	259		

**Table 4:** Final logistic regression model of factors associated with metabolic syndrome among adult HIV-infected patients, Riruta Health Centre, 2016

Factor	Adjusted Odds Ratio	95% C.I	*p-*value
Obesity (BMI > 30kg/m^2^)	5.37	2.80 – 10.27	< 0.001
Informal education	5.20	1.84 – 14.64	0.0018
Family history of hypertension	2.06	1.07 – 3.94	0.0299
Physical activity	0.28	0.13 – 0.60	0.0010

Eight factors were entered into the forward additive logistic regression model; obesity, physical activity, informal education, age, family history of hypertension, on antiretroviral treatment, informal employment and family history of cardiovascular disease

## Discussion

The study revealed that metabolic syndrome is prevalent among HIV-infected patients. The study prevalence is similar to that reported among HIV-infected patients using the same criteria in other countries; Australia (14%), Spain (16%), Barcelona (17%) and Ethiopia (21.1%) [13, 21, 24, 25]. The study prevalence was however much lower than the prevalence of metabolic syndrome in HIV-infected patients in Tanzania (25.6%), Denmark (27%), Cameroon (32.8%), Portugal (41.9%) and South Africa (60.6%) [19, 20, 22, 26, 27]. The study prevalence is also lower than the 34.6% and 25.6% prevalence reported among the general population living in urban centres in Kenya in 2012 and 2017 respectively [17, 18]. The presence of metabolic syndrome in HIV-infected patients puts them at an increased risk of developing cardiovascular disease and Type 2 diabetes [14, 15, 17]. This poses a higher risk of associated cardiovascular morbidity and mortality among HIV-infected patients in addition to opportunistic infections that they are predisposed to [24]. The study found that there were gender differences in prevalence of metabolic syndrome with a higher prevalence observed among female patients. Other studies have reported higher prevalence among females; 44.4 vs. 9.9% in Cameroon, and 25 vs. 12% in Australia [12, 17]. The observed higher prevalence among females in this population could be due to biological, hormonal and environmental factors that are thought to be contributing to occurrence of metabolic syndrome in women [1]. The prevalence was higher with increasing age and highest among those aged > 55 years. This observation has been reported in other similar studies conducted in Kenya, Tanzania, Ethiopia and Australia [18, 20, 21, 24]. The higher prevalence in older age-groups is supported by the fact that ageing has been shown to have a causal effect in metabolic syndrome [1]. Abdominal obesity, low HDL-cholesterol and hypertension were the most prevalent components in this urban population of HIV-infected persons. Abdominal obesity was present in over half while hypertension and low HDL-cholesterol in over one third of the study participants. Similarly, a study conducted among HIV-infected patients in Tanzania reported high prevalence of abdominal obesity (52.5%), low HDL-cholesterol (85.9%) and hypertension (49.2%) [20]. The high prevalence of these components suggests that there are many patients with precursors for metabolic syndrome. This presents an opportunity to delay development of cardiovascular disease and Type 2 diabetes if early cardiovascular risk assessment is offered with lifestyle modification for those identified to be at risk [1].

Abdominal obesity and low HDL-Cholesterol were more prevalent among the female patients while hypertriglyceridemia, hypertension and hyperglycaemia were more prevalent among the males. The prevalence of the individual components of metabolic syndrome by gender was consistent with figures reported in Tanzania and South Africa [20, 22]. Similarly, gender differences were also observed in an urban general population in Nairobi, Kenya [18]. The observed gender differences in the prevalence of the individual components in this population could be attributed to hormonal and biological factors [1]. Obesity, lack of formal education, ages > 55 years and family history of hypertension increased the likelihood of metabolic syndrome. Obesity is a known modifiable risk factor for metabolic syndrome [1, 28]. Having attained secondary or tertiary level education was found to be protective (OR = 0.3, P = 0.012) of metabolic syndrome in the general population in Kenya [18]. Ageing has been shown to have a causal effect in metabolic syndrome [1]. There was an increased likelihood of metabolic syndrome in older study participants drawn from the urban general population in Kenya [18]. Heredity and genetics have been shown to have a causal effect in metabolic syndrome [1]. The study also showed that physical activity and ART reduce likelihood of metabolic syndrome. Studies have shown that physical inactivity and a sedentary lifestyle predispose to metabolic syndrome [1, 29]. The finding on ART should be interpreted with caution since the number of patients not on ART (n = 5) in the study was small. A possible explanation for the small number of patients not on ART is that Kenya as a country has moved towards the test and treat approach where all HIV-infected patients are initiated on ART irrespective of the CD4 count. However, it is possible that the patients who were on ART in our study had received nutrition counselling and lifestyle modification advice during the health talks and psychosocial support group meetings at the treatment facility hence ART appears protective in this population. In contrast, other studies have demonstrated that ART leads to increased survival of HIV patients, improved quality of life and metabolic complications as adverse effects in patients receiving ART [22, 28, 30]. In another prospective cohort study, ART was shown to increase the prevalence of metabolic syndrome from 16.6% to 25% after 48 weeks of the study population receiving the treatment [13]. Our study had limitations. We could not establish causal association or the temporal sequence of events since this was a cross-sectional study. Fasting blood sugar and lipid profile testing was based on self-report of having fasted for 12 hours, there is possible misclassification of glycaemia and lipid profile abnormalities with potential to interpret random blood specimens as fasted states. However, this does not affect assessment of the prevalence of metabolic syndrome or factors associated with the syndrome among HIV-infected patients.

## Conclusion

Metabolic syndrome is prevalent among HIV-infected persons. The high prevalence of abdominal obesity, dyslipidemia and hypertension suggests that there are many HIV-infected patients with precursors for metabolic syndrome. Obesity, lack of formal education, family history of hypertension, and physical inactivity are associated with metabolic syndrome. Screening for risk factors, promotion of healthy lifestyle, and nutrition counselling should be offered routinely in HIV care and treatment clinics.

### What is known about this topic

- Metabolic syndrome is known to be prevalent among HIV infected persons in other countries with prevalence of up to 60% reported;
- People living with HIV are predisposed to metabolic syndrome due to the metabolic complications that are associated with long term use of antiretroviral treatment;
- Screening for non-communicable diseases is not prioritised in HIV care settings.

### What this study adds

- Screening for metabolic syndrome can be integrated into routine services offered at HIV care and treatment centres with minimal resources;
- Metabolic syndrome, abdominal obesity and hypertension is prevalent among the adult HIV-infected persons living in an urban town in Kenya with prevalence increasing with older ages;
- Metabolic syndrome in HIV is associated with level of education, family history of hypertension, obesity and physical activity.

## Competing interests

The authors declare no competing interests.
